# Patient characteristics and procedural variables are associated with length of stay and hospital cost among unilateral primary total hip arthroplasty patients: a single-center retrospective cohort study

**DOI:** 10.1186/s12891-022-06107-w

**Published:** 2023-01-04

**Authors:** Guoqing Li, Fei Yu, Su Liu, Jian Weng, Tiantian Qi, Haotian Qin, Yixiao Chen, Fangxi Wang, Ao Xiong, Deli Wang, Liang Gao, Hui Zeng

**Affiliations:** 1 Department of Bone & Joint Surgery, Peking University Shenzhen Hospital, Shenzhen, People’s Republic of China 518036; 2 National & Local Joint Engineering Research Center of Orthopaedic Biomaterials, Peking University Shenzhen Hospital, Shenzhen, People’s Republic of China 518036; 3 Center for Clinical Medicine, Huatuo Institute of Medical Innovation (HTIMI), 10787 Berlin, Germany; 4 Sino Euro Orthopaedics Network (SEON), Berlin, Germany

**Keywords:** Total hip arthroplasty, Patient characteristics, Procedural variables, Length of stay, Hospital cost

## Abstract

**Background:**

Total hip arthroplasty (THA) is a successful treatment for many hip diseases. Length of stay (LOS) and hospital cost are crucial parameters to quantify the medical efficacy and quality of unilateral primary THA patients. Clinical variables associated with LOS and hospital costs haven’t been investigated thoroughly.

**Methods:**

The present study retrospectively explored the contributors of LOS and hospital costs among a total of 452 unilateral primary THA patients from January 2019 to January 2020. All patients received conventional in-house rehabilitation services within our institute prior to discharge. Outcome parameters included LOS and hospital cost while clinical variables included patient characteristics and procedural variables. Multivariable linear regression analysis was performed to assess the association between outcome parameters and clinical variables by controlling confounding factors. Moreover, we analyzed patients in two groups according to their diagnosis with femur neck fracture (FNF) (confine THA) or non-FNF (elective THA) separately.

**Results:**

Among all 452 eligible participants (266 females and 186 males; age 57.05 ± 15.99 year-old), 145 (32.08%) patients diagnosed with FNF and 307 (67.92%) diagnosed with non-FNF were analyzed separately. Multivariable linear regression analysis revealed that clinical variables including surgery duration, transfusion, and comorbidity (stroke) among the elective THA patients while the approach and comorbidities (stoke, diabetes mellitus, coronary heart disease) among the confine THA patients were associated with a prolonged LOS (*P* < 0.05). Variables including the American Society of Anesthesiologists classification (ASA), duration, blood loss, and transfusion among the elective THA while the approach, duration, blood loss, transfusion, catheter, and comorbidities (stoke and coronary heart disease) among the confine THA were associated with higher hospital cost (*P* < 0.05). The results revealed that variables were associated with LOS and hospital cost at different degrees among both elective and confine THA.

**Conclusions:**

Specific clinical variables of the patient characteristics and procedural variables are associated the LOS and hospital cost, which may be different between the elective and confine THA patients. The findings may indicate that evaluation and identification of detailed perioperative factors are beneficial in managing perioperative preparation, adjusting patients’ anticipation, decreasing LOS, and reducing hospital cost.

## Background

Total hip arthroplasty (THA) is a successful and effective treatment in pain relief and functional restoration for various hip diseases, and it might constitute financial burden for the healthcare system [1]. Cases of elective THA might increase over the next decade as the elderly population grows and the impact from COVID-19 pandemic on the elective surgical operations lessens [2, 3]. Previous literature suggested that a substantial rise of THA should be addressed in health policies, and the diagnosis-related groups (DRGs) payment had potential cost-saving implications with possible higher efficacy and better use of the medical insurance [4, 5]. To meet the needs of ageing population with medical insurance burden, the establishment on the health care reform and the DRGs-based payment has been developed rapidly [6, 7]. New evidence implied that the focus of research has shifted to reducing the length of stay (LOS) and hospital cost as primary outcomes of success, which were associated but might be different with each other, with the aim to establish a better model for the patients [8].

Previous literature already classified the clinical variables possibly affecting the surgical outcomes into two categories including patient characteristics and procedure variables, however, definitive conclusions were not reached among the literatures [9]. Thus, further clarification and evaluation of the potential clinical variables associated with THA patients are still necessary. Moreover, for the benefit of local patients, to determine region-specific relevant risk factors including patient characteristics and procedure variables is essential for specific hospitals as they may vary among institutions.

The present study intended to investigate the possible impacts of available clinical variables on the LOS and hospital cost of THA patients in our institute and report the THA-relevant economic status in China. We hypothesize that specific patient characteristics and procedural variables are associated with the LOS and hospital cost among unilateral primary THA patients.

## Methods

### Participants

**Fig. 1:**
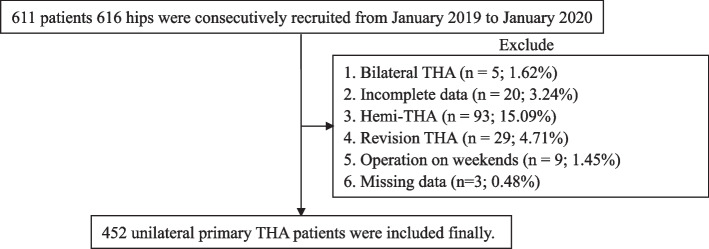
Flowchart illustrating patients selection

The study was conformed to Declarations of Helsinki and approved by Ethics Committee of Peking University Shenzhen Hospital (No.2020013), which was carried out from January 2019 to January 2020 in Peking University Shenzhen Hospital, a modernized and comprehensive general public hospital. The clinical data of the unilateral primary THA patients were retrospectively analyzed with patients’ consent, and the selection process of patients was illustrated in Fig. 1. Patients were excluded, if they had incomplete data, refused to be discharged till the stitches were removed after the incision healing, or underwent other procedures (e.g., bilateral THA, hemi-THA, or revision arthroplasty). Patients who underwent THA on weekends were also excluded because the LOS might be affected by the surgery timing [10]. For those unilateral primary THA patients, a standardized surgical intervention was performed with an anterior-lateral approach (ALA) or a posterior lateral approach (PLA) and individualized according to their diagnosis and conditions [11]. All the THA were conducted by three senior surgeons who were fellowship-trained and performed over 200 THA annually.

The postoperative patients then received personally tailored rehabilitation services from the rehabilitation experts within our department, rather than transferring to the rehabilitation department or hospital. Of note, per our standard protocol, we applied a conventional rehabilitation program for these patients, instead of the Enhanced Recovery After Surgery (ERAS) [12].

### Clinical variables and outcome parameters

Available clinical variables (patient characteristics and procedural variables) were reviewed and collected from the electronic medical record (EMR) retrospectively. The patient characteristics including age, gender, body mass index (BMI), and American Society of Anesthesiologists classification (ASA) of each patient were recorded. Their diagnosis was categorized as hip osteoarthritis (OA), rheumatoid arthritis (RA), ankylosing spondylitis (AS), avascular necrosis (AVN), developmental dysplasia of the hip (DDH), and femoral neck fracture (FNF). Patients were separated into the confine THA (for FNF) and elective THA (for non-FNF) due to the characteristics of the THA [13]. Relevant available comorbidities from the EMR included hypertension, diabetes metabolic (DM), coronary heart disease (CHD), stroke, and others (e.g., bleeding disorders, chronic obstructive pulmonary disease, chronic kidney disease, anemia, and dyspnea).

Procedural variables were defined as follows: Anesthesia type was categorized as general or regional [14]; Blood loss was calculated as patient blood volume (PBV, ml) × (preop-hematocrit - postop-hematocrit) [15]. PBV = K1 × height^3^ (m) + K2 × mass (kg) + K3, (Male: K1 = 0.3669, K2 = 0.03219, K3 = 0.6041; Female: K1 = 0.3561, K2 = 0.03308, K3 = 0.1833) [16]; Duration of operation was defined as time from incision to the end of all procedures completed; Urinary catheter and incision drainage decisions were depended on surgeons’ preference and patients’ conditions [17].

The outcome parameters were LOS and hospital cost. The LOS was defined as the period between the admitted date and the discharge date [18]. The hospital cost covered all the payments for drugs, nursing, treatment, and examination during the perioperative period.

### Discharge criteria

Even the discharge criteria varied worldwide [19], the discharge criteria in our institute were hip joint flexion of 90 degrees, stable vital signs and normal laboratory values, sufficient ability to stand and walk safely with or without aids, sufficient ability to walk up and down stairs without assistance, sufficient ability to perform personal care, adequate knowledge of activity restrictions and wound care, satisfactory pain control, and personal acceptance of discharge. Generally, the discharged patients report the pain visual analogue scale ≤3 and the muscle strength grade ≥ 4 [20].

### Statistical analysis

The distribution of data was evaluated with the Kolmogorov-Smirnov test. Normally distributed continuous variables were summarized by mean ± standard deviation (SD) while non-normally distributed continuous variables were expressed with median (interquartile range). Categorical variables were summarized by frequency and percentage. The baseline characteristics of participants were compared. Student *t-test* was applied for normally distributed continuous variables while Mann-Whitney U test was performed for non-normally distributed continuous variables. Less than 1% of patients with missed information were deleted finally. We performed multivariable liner regression analysis to assess the association in LOS and hospital cost with patient characteristics and medical variables. The model was adjusted for gender, age, BMI, diagnosis, comorbidity, ASA, approach, duration, anesthesia, blood loss, transfusion, catheter, and drainage. Patients were analyzed in two groups per diagnosis as FNF (confine THA) and non-FNF (elective THA) separately. The results were displayed in the forest plots. Results are presented as regression coefficients, in companies with 95% confidence intervals (CIs) and *P* values. The level of significance was set at a 2-sided level of 0.05. All statistical analysis was performed on R version 4.0.2 (R Foundation for Statistical Computing, Vienna, Austria).

## Results

Four hundred fifty-two (452) eligible participants were finally included (266 females and 186 males, aged 57.05 ± 16.00 years old; BMI 22.86 ± 3.33 kg/m^2^). Patients’ demographic and procedure variables were listed in Tables and details were described as follows.

Table 1 summarized the demographic characteristics of the study group population. Participants were diagnosed with FNF (32.08%), AVN (30.09%), hip OA (6.19%), AS (12.17%), and DDH (19.47%) for THA. Most patients were evaluated as ASA I & II (94.47%) while some of them were classified as ASA III (5.53%). The LOS was 16.29 ± 5.28 days while the hospital cost was 13,940.33 ± 3928.75 U.S. Dollars (USD).

**Table 1:** Characteristics of unilateral primary THA patients (*n =* 452)

Characteristics	Values
**Gender**^**a**^	
Female	266 (59.00%)
Male	186 (41.00%)
**Age**^**b**^ **- years**	57.05 ± 16.00
**BMI**^**b**^ **- kg/m**^**2**^	22.86 ± 3.33
**Diagnosis**^**a**^	
HOA	28 (6.19%)
AS	55 (12.17%)
AVN	136 (30.09%)
DDH	88 (19.47%)
FNF	145 (32.08%)
**Comorbidity**^**a**^	
None	153 (33.85%)
Hypertension	55 (12.17%)
DM	15 (3.32%)
CHD	44 (9.73%)
Stroke	35 (7.74%)
Others	150 (33.19%)
**ASA classification**^**a**^	
I	172 (38.05%)
II	255 (56.42%)
III	25 (5.53%)
**Total LOS**^**b**^ **- days**	16.29 ± 5.28
**Hospital costs**^**b**^ **- USD**	13,940.33 ± 3928.75

Abbreviations: *AS* Ankylosing spondylitis, *AVN* Aseptic vascular necrosis, *ASA* American Society of Anesthesiologists, *BMI* Body mass index, *CHD* Coronary heart disease, *DDH* Developmental dysplasia of the hip, *DM* Diabetes metabolic, *FNF* Femoral neck fracture, *HOA* Hip osteoarthritis, *LOS* Length of stay, *USD* US dollars, *n* Number

^a^The values of categorical statistics are given as the number and percentage (%) of patients

^b^The values of continuous statistics are given as the mean and the standard deviation

Table 2 summarized the procedural variables of unilateral primary THA patients. Patients underwent the THA via either the PLA (81.86%) or ALA (18.14%). The duration of the surgical procedure ranged from 60 to 190 minutes (135.73 ± 29.21). The majority of patients were operated under the spinal or epidural anesthesia (82.74%). Blood loss was evaluated as 300.35 ± 175.00 ml and the transfusion were recorded as 0.77 ± 1.41 units. Patients were catheterized temporarily (87.83%) during surgery and used the drainage (35.40%).

**Table 2:** Procedural variables of unilateral primary THA patients (*n =* 452)

Variables	Values
**Approach** ^**a**^	
ALA	82 (18.14%)
PLA	370 (81.86%)
**Duration** ^**b**^ **- minutes**	135.73 ± 29.21
**Anesthesia** ^**a**^	
General	78 (17.26%)
Regional	374 (82.74%)
**Blood loss** ^**b**^ **- ml**	300.35 ± 175.00
**Transfusion** ^**b**^ **- unit**	0.77 ± 1.41
**Catheter** ^**a**^	
No	55 (12.17%)
Yes	397 (87.83%)
**Drainage** ^**a**^	
No	292 (64.60%)
Yes	160 (35.40%)

Abbreviations: ALA, anterior lateral approach; PLA, posterior lateral approach; n, number

^a^ The values of categorical statistics are given as the number and percentage (%) of patients

^b^ The values of continuous statistics are given as the mean and the standard deviation

**Fig. 2:**
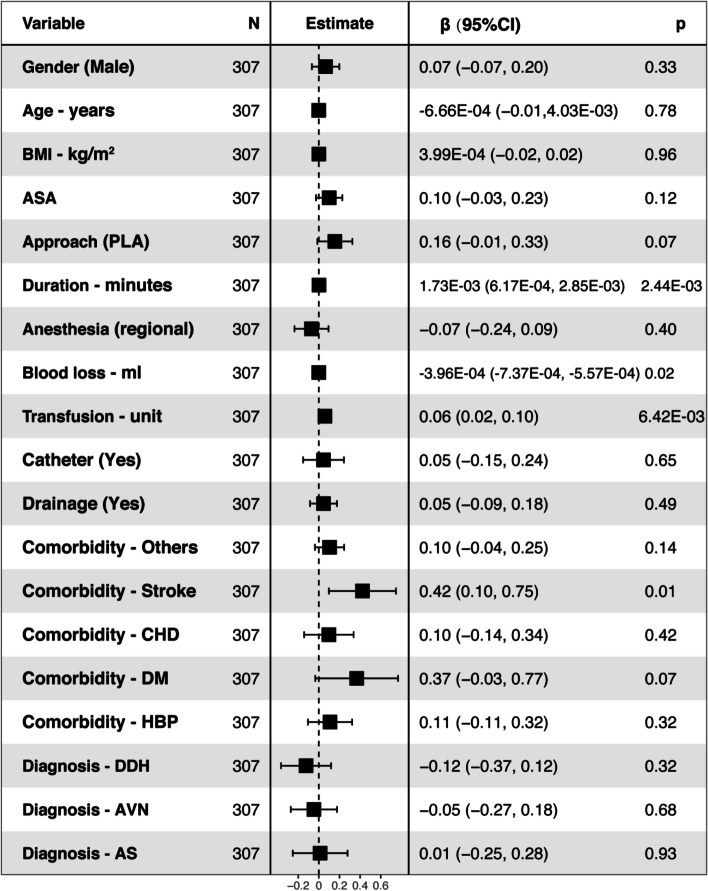
Factors associated with LOS among the elective THA by multivariable linear regression analysis (R^2^ = 0.1422, intercept = 3.49)

**Fig. 3:**
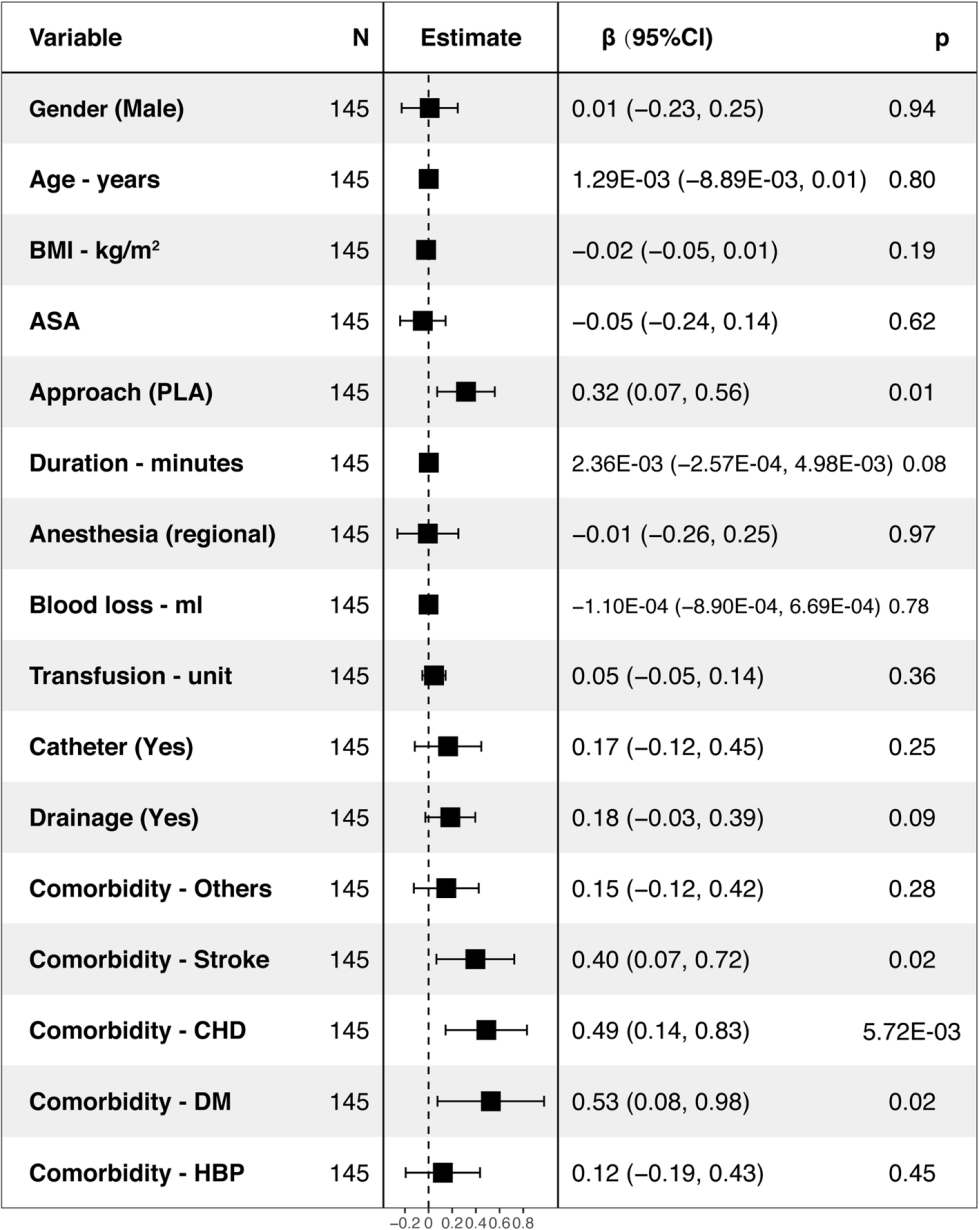
Factors associated with LOS among the confine THA by multivariable linear regression analysis (R^2^ = 0.1364, intercept = 3.52)

Multivariable linear regression analysis revealed the association between the clinical variables and outcome parameters (LOS and hospital cost). The results were displayed in forest plots. For the elective THA (R^2^ = 0.1422, intercept = 3.49), the surgery duration (95% CI: 6.17E-04, 2.85E-03), transfusion (95% CI: 0.02, 0.11), and comorbidity of stroke (95% CI: 0.10, 0.75) were associated with the prolonged LOS while the blood loss (95% CI: -7.37E-04, − 5.57E-05) was associated with the shorter LOS (*P* < 0.05) (Fig. 2). For the confine THAs (R^2^ = 0.1364, intercept = 3.52), the approach (95% CI: 0.07, 0.56) and comorbidities (stroke (95% CI: 0.07, 0.72), CHD (95% CI: 0.14, 0.83), and DM (95% CI: 0.08, 0.98)) were associated with the prolonged LOS (*P* < 0.05) (Fig. 3).

**Fig. 4:**
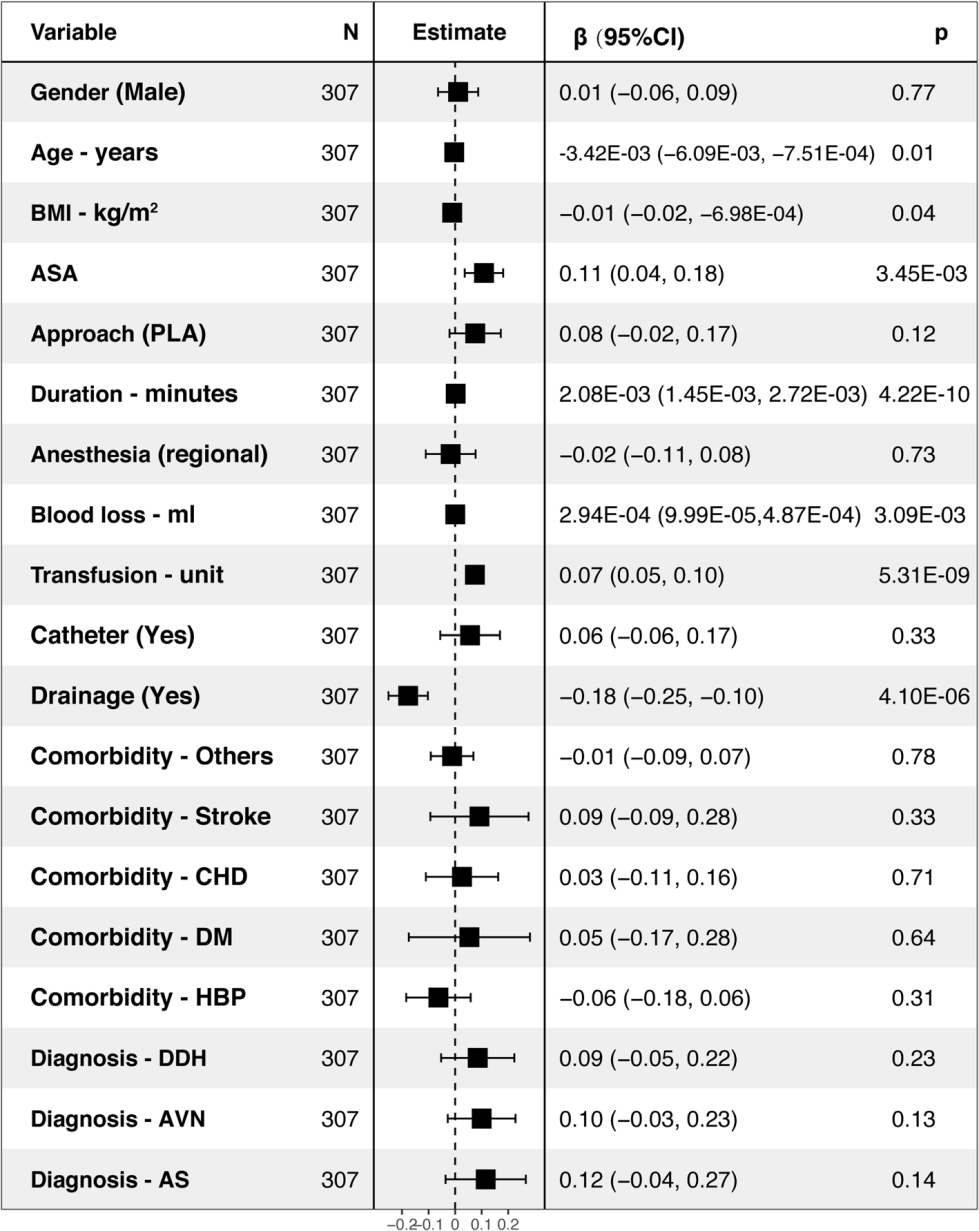
Factors associated with hospital cost among the elective THA by multivariable linear regression analysis (R^2^ = 0.3946, intercept =16.13)

**Fig. 5:**
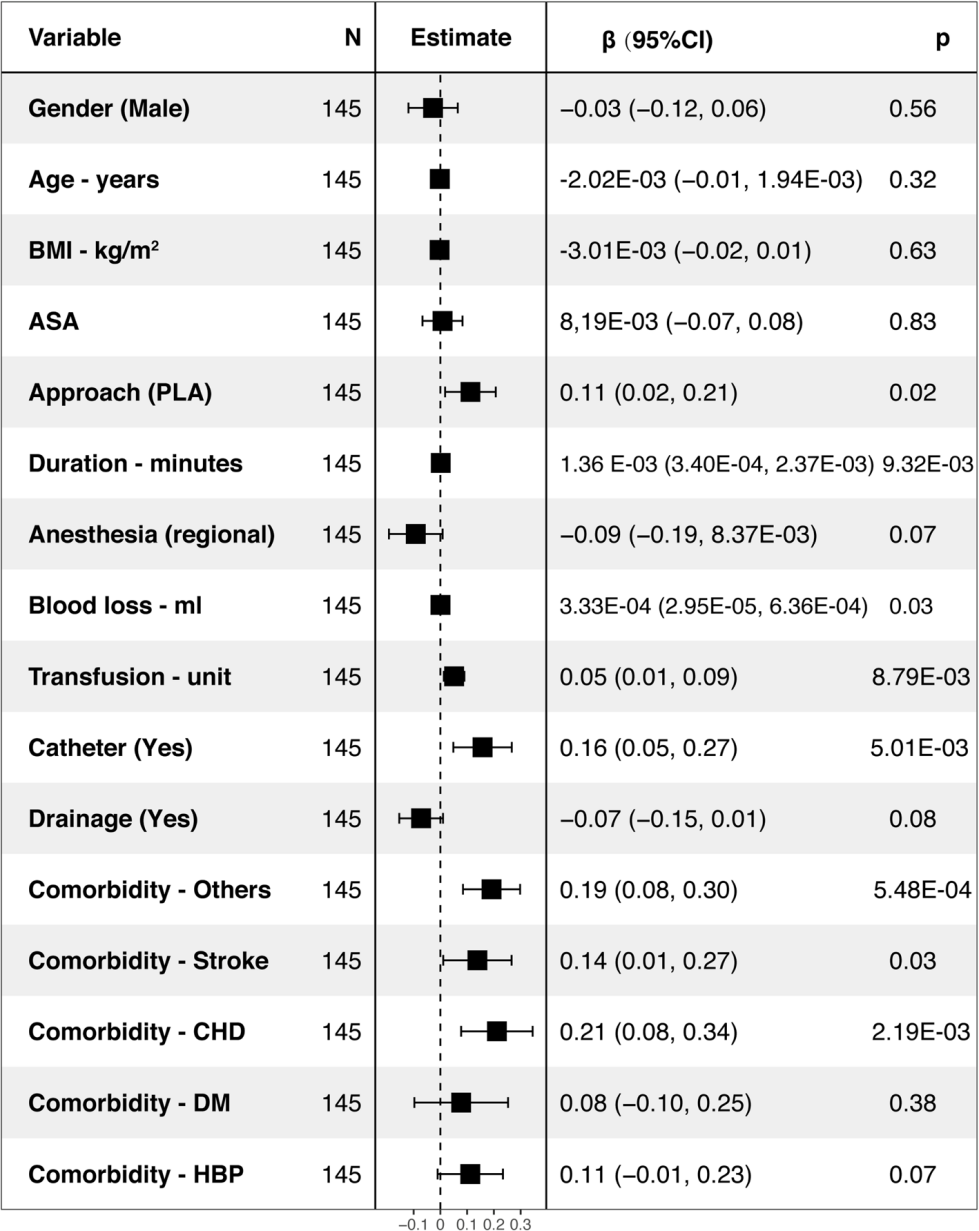
Factors associated with hospital cost among the confine THA by multivariable linear regression analysis (R^2^ = 0.2949, Intercept = 15.96)

The ASA (95% CI: 0.04, 0.18), surgery duration (95% CI: 1.45E-03, 2.72E-03), blood loss (95% CI: 9.99E-05, 4.87E-05), and transfusion (95% CI: 0.05, 0.10) were associated with higher hospital cost, while the age (95% CI: -6.09E-03, − 7.51E-04), BMI (95% CI: − 0.02, − 6.98E-04), and drainage use (95% CI: − 0.25, − 0.10) were associated with lower hospital cost (*P* < 0.05) by multivariable linear regression analysis among the elective THA (R^2^ = 0.3946, intercept =16.13) (Fig. 4). For the confine THA (R^2^ = 0.2949, Intercept = 15.96), the approach (95% CI: 0.02, 0.21), duration (95% CI: 3.40E-04, 2.37E-03), blood loss (95% CI: 2.95E-05, 6.36E-04), transfusion (95% CI: 0.01, 0.09), catheter (95% CI: 0.05, 0.27), comorbidities (stroke (95% CI: 0.01, 0.27), CHD (95% CI: 0.08, 0.34) and others (95% CI: 0.08, 0.30)) were associated with higher hospital cost (*P* < 0.05) (Fig. 5).

## Discussion

The key finding of the present study was that the comorbidities, longer operation duration, and post-operative transfusion might be associated with the prolonged LOS significantly. The ASA II, longer duration, more blood loss, higher transfusion rate, and usage of the catheter might be associated with increased hospital cost for unilateral primary THA patients. Identifying these patient characteristics and refining the procedure variables might benefit specific patients and ultimately improve the medical efficiency and quantity.

The longer duration, more blood loss, and higher transfusion rate were associated with extended LOS and increased hospital cost. Our data showed that the longer duration was usually accompanied with more blood loss and the need for transfusion, which were in line with previous reports that improving surgical techniques could reduce the risk of adverse outcomes [21]. THA with extensive bleeding and demand for transfusion not only yields an economic burden on the health care system but also increases the risk of perioperative adverse events. The perioperative blood management, as a multi-disciplinary approach, should be designed and applied to identify high-risk patients, reduce postoperative complications, improve the resource allocation, and optimize the ultimate patient care [22, 23]. Advancing the THA surgical techniques by simplifying procedure protocols and improving surgery accuracy is clinically necessary and also beneficial to improve the postoperative prognosis.

Intraoperative catheterization was associated with increased hospital cost. Previous evidence preferred no routine use of catheterization to avoid the urinary retention or infection [24]. The incidence of postoperative urinary retention, as a common postoperative complication after joint arthroplasties, ranges from 4.10 to 46.3% [25]. Patients with a history of urinary retention and high volume of fluid tend to experience urinary retention and infection postoperatively [26]. Generally, the catheters should be removed within 48 hours postoperatively or no catheterization intraoperatively is recommended. Intermittent catheterization or removing it in the early stage would be better choices.

Drainage was associated with prolonged LOS. Drainage could result into complications due to the restriction of the early mobilization postoperatively [27, 28]. However, to remain the drainage or not has no adverse impact on the blood loss or functional recovery if it is pulled out in time [29, 30]. We recommend using the drainage in accord with the personal situation of the patients, and a proper usage of drainage might contribute to the cost reduction. No drainage for easy THA may be a better choice but it should be further evaluated in detail in complex THA.

Comorbidities were associated with extended LOS. Comorbidities as no-modifiable factor may affect the nutrition and result into the poor clinical status [31]. Additionally, investigations revealed that younger patients with better preoperative status tended to achieve better long-term postoperative improvements [32, 33]. Therefore, multiple disciplinary discussions and perioperative evaluation as well as appropriate preventive measures are necessary to management those comorbidities [34]. Positive interventions should also be prepared for those patients with specific comorbidities during the perioperative management.

Previous analyses also demonstrated that medical efficacy and quality were independently associated with patient demographic [35], which were approved in the current study. Morbid obesity was treated as one of the risk factors and associated with periprosthetic joint infection [36], but the infection risk generally could be mitigated with proper treatment timely. Our study revealed that body mass index (BMI) was associated with reduced hospital cost, which might be due to the sample size. Understanding current anesthesia practice pattern might be good aimed at maximizing effective postoperative pain control [37]. Lower preoperative albumin and abnormal hematological tests were risk factors for predicting adverse outcomes following the primary THA [38]. These findings may not only direct surgeons to devote more attention to these relevant clinical variables associated with LOS and hospital cost, but also guide patients’ expectations during the surgery consultation and perioperative management.

Of note, the LOS in the present study is much longer than other reports in the literature. There are two possible reasons for this deviation. The first reason is the unapplied ERAS in our institute for THA patients reported in this study. Our data showed the LOS ranged 16.29 ± 5.28 days, which is indeed longer than the studies [39–41] in the literature with patients treated with ERAS [42, 43] but is in line with other studies with patients not treated with ERAS from both China (around 15 to 20 days) [44, 45] and oversea hospitals (up to 28 days) [46]. The second reason is that the integrated in-house rehabilitation services, applied for the patients prior to discharge within our institute, extend the LOS of our patients compared to the LOS of those patients (after their discharges) transferred again to a specific rehabilitation department or hospital. Therefore, the LOS in our setting is not notably longer than in other jurisdictions from these perspectives.

The present study has several limitations. First, no casual relationships could be demonstrated in this observational and retrospective study. Second, even though the standard of discharge may be varied among institutions, the standardized discharge criteria used in this study were also well accepted by other Chinese hospitals, performing the in-house orthopedic rehabilitation services for the patients. Moreover, the patients in the present study received conventional in-house rehabilitation services (rather than the ERAS) within our institute prior to discharge, which extends their LOS and yields higher hospital costs. Therefore, our data should be interrupted with caution, especially for those institutes where the rehabilitation protocols are much different from ours. Thirdly, due to the limitation of our dataset and the retrospective nature of this study, we could not obtain all potential patient-relevant clinical variables, which may be the reason for the low R^2^ for LOS and the weak (nonlinear) association between the LOS and currently available factors. Therefore, the additional nonlinear prediction would be considered in our future study. Finally, further prospective studies with more robust experimental designs and larger sample sizes are necessary to confirm the results of this study.

## Conclusions

Our study demonstrates that patient characteristics and procedure variables might affect the medical efficacy and quality in terms of the LOS and hospital cost for unilateral primary THA patients. The impacts of these clinical variables are different for the elective and confine THA patients. The preliminary data from this study hint that the possible clinical benefits could be achieved if the practitioners precautiously identify surgical candidates at specific risk levels and modify specific clinical variables.

## Data Availability

The data are not publicly available due to them containing information that could compromise research participant privacy or consent but are available from the corresponding author on reasonable request with the permission of Department of bone and joint in Peking University Shenzhen Hospital.

## Abbreviations

THA: Total hip arthroplasty
LOS: Length of stay
DRGs: Diagnosis-related groups
ALA: Anterior-lateral approach
PLA: Posterior lateral approach
ERAS: Enhanced recovery after surgery
BMI: Body mass index
ASA: American Society of Anesthesiologists classification
HOA: Hip osteoarthritis
RA: Rheumatoid arthritis
AS: Ankylosing spondylitis
FNF: Femoral neck fracture
AVN: Avascular necrosis
DDH: Developmental dysplasia of the hip
EMR: Electronic medical record
DM: Diabetes metabolic
CHD: Coronary heart disease
PBV: Patient blood volume
SD: Standard deviation
CI: Confidence intervals
VIF: Variance inflation factor
USD: U.S. Dollar
